# Supplementary material for: Reconstruction of plant–pollinator networks from observational data
Source: Nat Commun. 2021 Jun 23;12:3911. doi: 10.1038/s41467-021-24149-x (PMC8222257; doi:10.1038/s41467-021-24149-x)
Supplement: Supplementary file 1 — Supplementary Information [file 41467_2021_24149_MOESM1_ESM.pdf]

Reconstruction of plant–pollinator networks from observational data

Jean-Gabriel Young,[1,2,3,*] Fernanda S. Valdovinos,[3,4,5,†] and M. E. J. Newman[3,6,‡]

[1] Department of Computer Science, University of Vermont, Burlington, Vermont, USA
[2] Vermont Complex Systems Center, University of Vermont, Burlington, Vermont, USA
[3] Center for the Study of Complex Systems, University of Michigan, Ann Arbor, Michigan, USA
[4] Department of Environmental Science and Policy, University of California, Davis, California, USA
[5] Department of Ecology and Evolutionary Biology, University of Michigan, Ann Arbor, Michigan, USA
[6] Department of Physics, University of Michigan, Ann Arbor, Michigan, USA

SUPPLEMENTARY NOTE 1. CONSISTENCY ON SYNTHETIC DATA

To demonstrate that recovery is possible in ecologically relevant scenarios, we carry out a simple recovery test, using synthetic data created with known parameters.

A. General procedure

Our procedure goes as follows. Given a known incidence matrix $\boldsymbol{B}$ and known parameters $(\boldsymbol{B}, C, r, \sigma, \tau)$, we first generate an artificial data set $\boldsymbol{M}$ using the likelihood

$$P(\boldsymbol{M}|\boldsymbol{B}, C, r, \sigma, \tau) = \prod_{ij} \frac{[C\sigma_i\tau_j(1 + rB_{ij})]^{M_{ij}}}{M_{ij}!} e^{-C\sigma_i\tau_j(1+rB_{ij})}. \tag{1}$$

We then use this data set $\boldsymbol{M}$ as input for the full inference procedure detailed in the main text. Our procedures returns parameter samples, and samples of the connection matrix $\boldsymbol{Q}$. We use these samples to compute estimates of the parameters by taking the sample mean. To get an estimate of $\boldsymbol{B}$, we set $\hat{B}_{ij} = 1$ if $\langle Q_{ij}\rangle > 0.5$ and $\hat{B}_{ij} = 0$ otherwise.

Once we have our estimates, we quantify how close they are to the true generating parameters with the following error measures:

- **Network**: the relative Hamming distance $\frac{1}{n_p n_a}||\boldsymbol{B} - \hat{\boldsymbol{B}}||_0$ between the true and estimated incidence matrix.
- **Abundances**: the Jensen-Shannon distance between σ and $\hat{\sigma}$, defined as $\sqrt{\frac{1}{2}[D(\sigma||m) + D(\hat{\sigma}||m)]}$, where $D(x||y)$ is the Kullback-Leibler divergence of distribution x and y, and where m is the distribution obtained by averaging σ and $\hat{\sigma}$, as $m_i = \frac{1}{2}(\sigma_i + \hat{\sigma}_i)$.
- **Preferences**: the relative error on r, defined as $|\hat{r} - r|/r$.

All of these quantities decrease as the fit becomes better.

B. Parametrization

The parameter space of our model is vast, so we restrict ourselves to ecologically relevant parametrization to better explore the relevant space thoroughly. By ecologically relevant we mean: (i) a small network, generally with more pollinators than plants; (ii) relatively sparsely connected; (iii) skewed distributions of abundances; (iv) a realistic sampling effort, such that $\sum_{ij} M_{ij}$ is on the order of a few hundreds, our a thousand at most; and (v) strong preferences, with $r \gg 1$. We consider various scenarios for each axis of the model, and create a series of experimental conditions by considering all combinations of these scenarios.

* jean-gabriel.young@uvm.edu
† fvaldovinos@ucdavis.edu
‡ mejn@umich.edu

1. Networks

We investigate two experimental conditions for the network structure $\boldsymbol{B}$.

In the first experimental condition, we generate networks directly from the prior

$$P(\boldsymbol{B}|\rho) = \prod_{ij} (1-\rho)^{1-B_{ij}} \rho^{B_{ij}}. \tag{2}$$

To match empirical conditions we investigate networks of sizes 10×20, 10×30, 20×40 and 20×60, which allows us to test the effect of the system size, and of the "aspect ratio." In all experiments we use $\rho = 0.15$ and modify the prior slightly to reject samples that contain a node of degree 0. This last step skews the expected density upward somewhat, but not by much since rejections are rare.

In the second experimental condition, we construct a realistic network by thresholding the empirical observation matrix $\boldsymbol{M}$ gathered at the Trois-Frères site of the Kaiser-Bunbury study [1]. We consider that there is an edge between two species i and j when $M_{ij} \geq 1$. Recall that we do not advocate this procedure when it comes to estimating the network from data, but it suffices for the purpose of testing our procedure.

2. Abundances

To generate distributions of abundances σ and τ, we use a two step process, where we first generate strengths vectors $\tilde{\sigma}$ and $\tilde{\tau}$ for the species, whose entries are not forced to sum to 1. We then use these strengths as the parameters of a Dirichlet distribution, from which we draw abundance distributions σ and τ. In this generative process, uniform strength vectors lead to roughly uniform abundance distributions. And conversely, heterogeneous strength vectors lead to heterogeneous distributions of abundances. Since it is generally accepted that abundances are skewed in plant-pollinator systems, we draw the strengths i.i.d. from a log-normal distribution whose underlying normal distribution has mean 0. For both the plants and the pollinator, we then consider two scenarios: (i) a standard deviation of $s = 1$ for the underlying distribution, leading to highly skewed strength and abundance, and (ii) a standard deviation of $s = 0.1$, leading to skewed, but more homogeneous abundances.

3. Other parameters

The other key parameters are r, the preference, and C, the sampling effort. We consider a scenario where interactions are not very specific, with $r = 10$, and a scenario where preferences are strong, $r = 100$. In our study of empirical networks, we additionally show result at $r = 50$, which roughly correspond to the value of r estimated in the main text. Finally, we test a wide range of sampling effort $C = [2, 5, 10, 20, 30, 40, 50, 100, 500]$. On networks of size and density comparable to empirically observed networks, these parameters lead to observation counts $\sum_{ij} M_{ij}$ similar to what we find in empirical data sets—a few hundred observations.

C. Results

The results our tests are shown in Fig. 1–3 In all cases, without exception, increasing the sampling effort leads to a more accurate reconstruction. As expected, the recovery is more precise for all parameters when the preference is large, say $r = 50$ or 100, because the data $\boldsymbol{M}$ then clearly separates the connected plant-pollinators pair from the disconnected ones. We note that while the recovered parameters are quite noisy at low values of C, the estimates becomes accurate when C is large enough to generate data sets with observations counts $\sum_{ij} M_{ij}$ on the order of a few hundred. We also find that recovery is equally accurate, regardless of whether the network is generated from the prior or using empirical data.

At insufficient sampling levels, say $M < 100$, we find that imperfect recovery is possible when $r = 50$ or 100 (such values of the preference are more realistic than $r = 10$, which we never found in any of experiments with empirical data sets). For instance, we find that the error on r is at most of 50%. The Hamming distance is always much lower than its maximal value (roughly 0.70 in the ER experiment, corresponding to the case where one completely mistakes edges for the absence of edges, and vice-versa). Finally, the Jensen-Shannon distance also remains low, compared to its maximal value of 1.

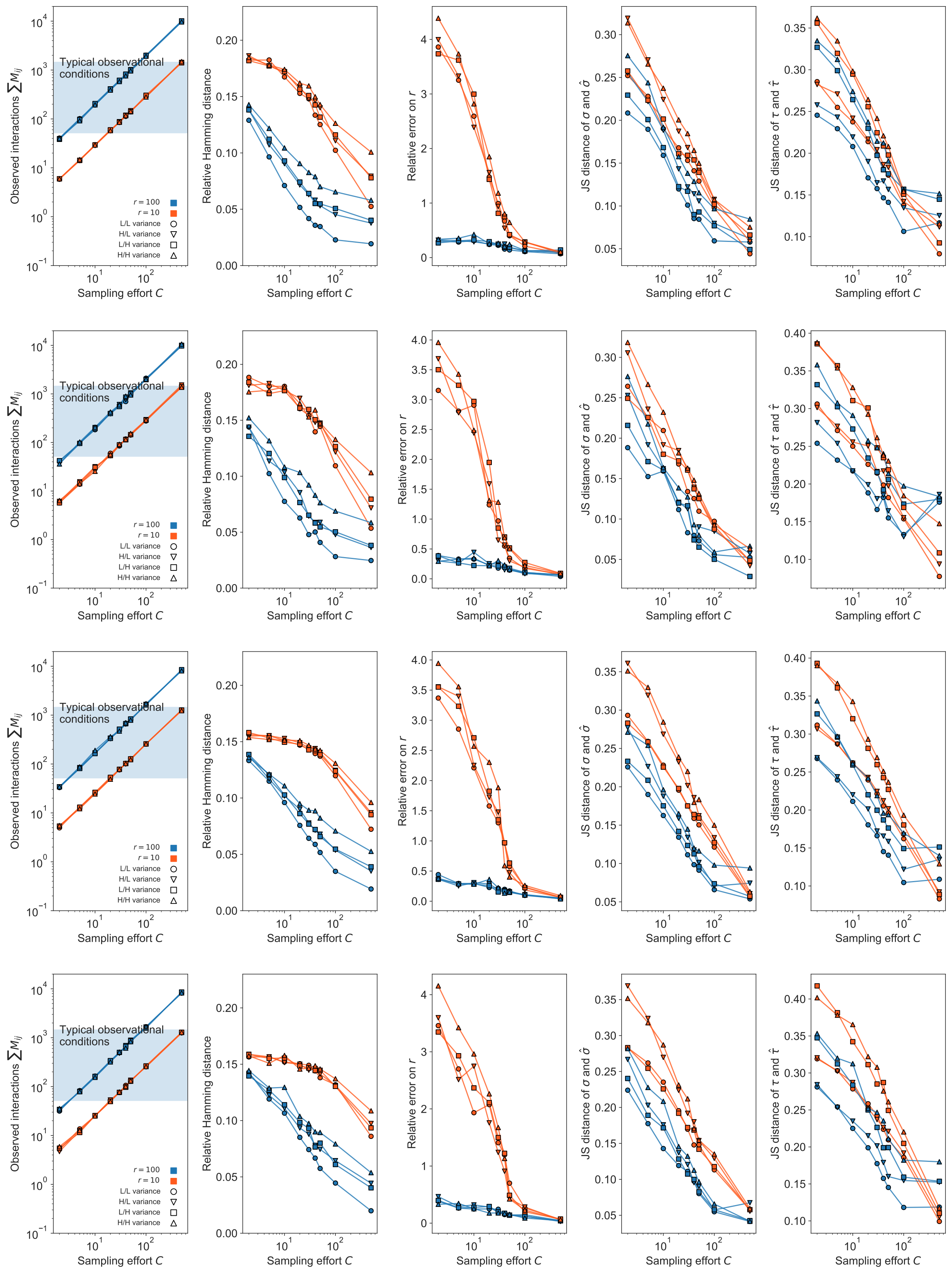

SUPPLEMENTARY FIGURE 1. Results of the reconstruction, for networks generated from the prior. Each set of results corresponds to a different combinations of preference r value (color) and of variances for the abundances distributions (indicated by symbols). Each row corresponds to a different set of network dimensions. From top to bottom, these networks are of dimensions: 10×20, 10×30, 20×40, 20×60. The mean density if of $\rho = 0.15$. Results are averaged over 80 realization of the data set instantiation and sampling procedure.

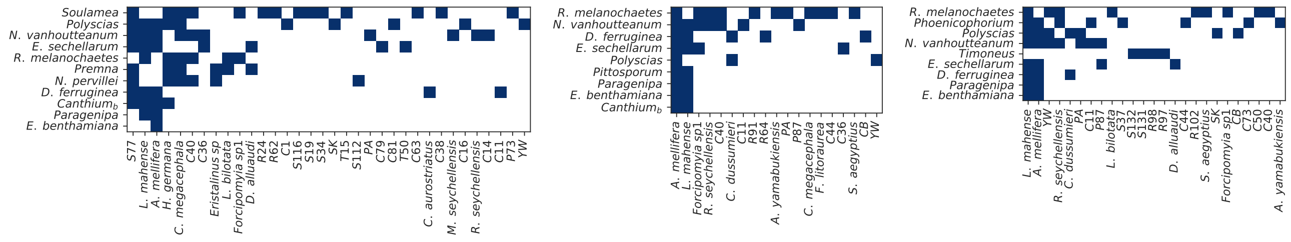

SUPPLEMENTARY FIGURE 2. Network structure used as input of our second set of tests. These incidence matrices are constructed by thresholding the data matrix sampled at the Trois-Frère site, in January, February and March 2012.

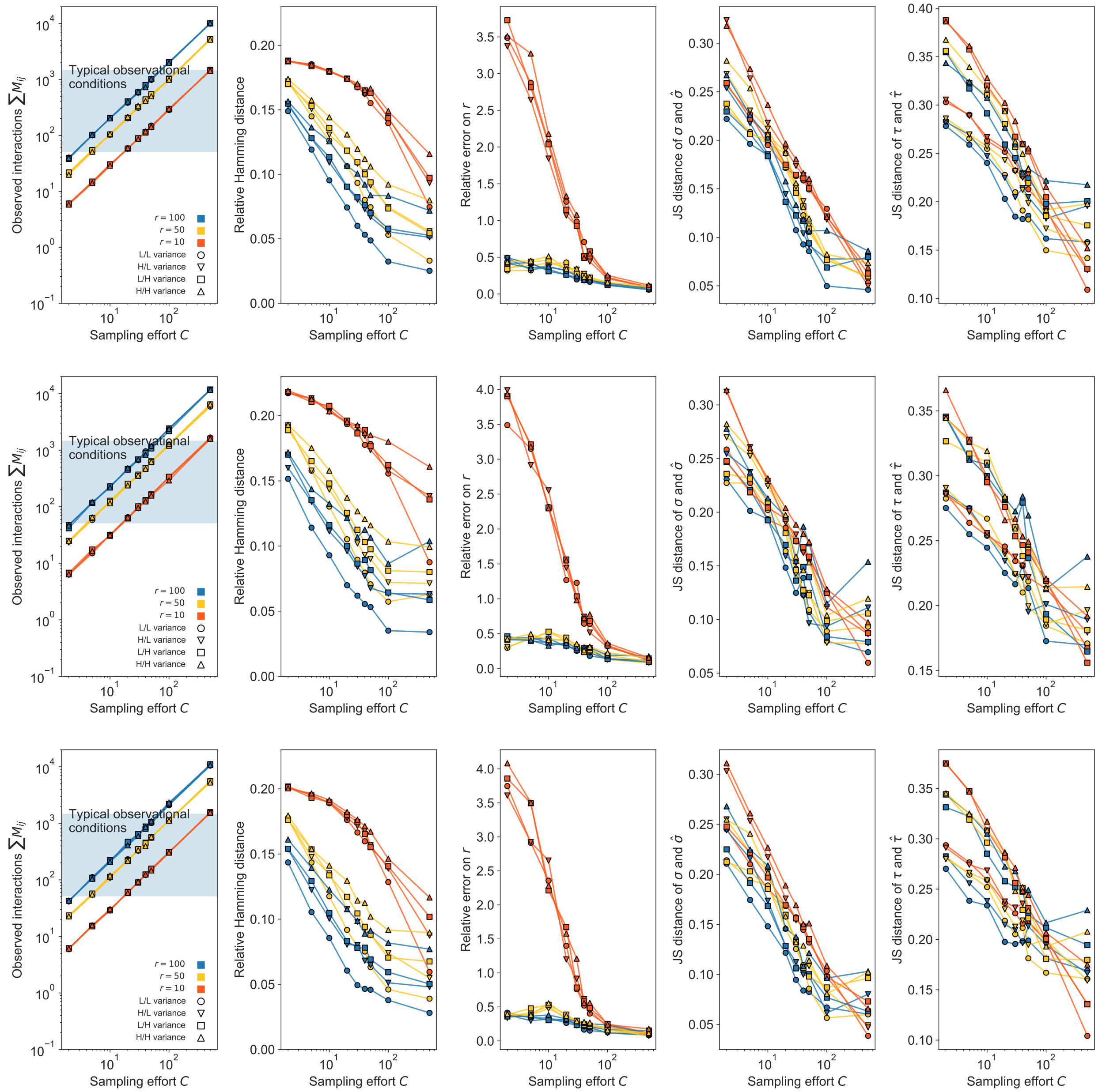

SUPPLEMENTARY FIGURE 3. Results of the reconstruction, for the networks shown in Fig. 2. Each set of results corresponds to a different combinations of preference r value (color) and of variances for the abundances distributions (indicated by symbols). Each row corresponds to a different network which are, from top to bottom: January 2012, February 2010 and March 2012. Results are averaged over 90 realization of the data set instantiation and sampling procedure.

SUPPLEMENTARY REFERENCES

[1] Christopher N Kaiser-Bunbury, James Mougal, Andrew E Whittington, Terence Valentin, Ronny Gabriel, Jens M Olesen, and Nico Blüthgen, "Ecosystem restoration strengthens pollination network resilience and function," Nature **542**, 223–227 (2017).
