# Supplementary material for: Reconstruction of plant–pollinator networks from observational data
Source: Nat Commun. 2021 Jun 23;12:3911. doi: 10.1038/s41467-021-24149-x (PMC8222257; doi:10.1038/s41467-021-24149-x)
Supplement: Supplementary file 2 — Reporting Summary [file 41467_2021_24149_MOESM2_ESM.pdf]

nature research

Corresponding author(s): Young, Jean-Gabriel

Last updated by author(s): May 13, 2021

Reporting Summary

Nature Research wishes to improve the reproducibility of the work that we publish. This form provides structure for consistency and transparency in reporting. For further information on Nature Research policies, see our Editorial Policies and the Editorial Policy Checklist.

Statistics

For all statistical analyses, confirm that the following items are present in the figure legend, table legend, main text, or Methods section.

n/a	Confirmed	
☐	☒	The exact sample size (*n*) for each experimental group/condition, given as a discrete number and unit of measurement
☐	☒	A statement on whether measurements were taken from distinct samples or whether the same sample was measured repeatedly
☒	☐	The statistical test(s) used AND whether they are one- or two-sided<br>*Only common tests should be described solely by name; describe more complex techniques in the Methods section.*
☐	☒	A description of all covariates tested
☐	☒	A description of any assumptions or corrections, such as tests of normality and adjustment for multiple comparisons
☐	☒	A full description of the statistical parameters including central tendency (e.g. means) or other basic estimates (e.g. regression coefficient) AND variation (e.g. standard deviation) or associated estimates of uncertainty (e.g. confidence intervals)
☒	☐	For null hypothesis testing, the test statistic (e.g. *F*, *t*, *r*) with confidence intervals, effect sizes, degrees of freedom and *P* value noted<br>*Give P values as exact values whenever suitable.*
☐	☒	For Bayesian analysis, information on the choice of priors and Markov chain Monte Carlo settings
☒	☐	For hierarchical and complex designs, identification of the appropriate level for tests and full reporting of outcomes
☒	☐	Estimates of effect sizes (e.g. Cohen's *d*, Pearson's *r*), indicating how they were calculated

Our web collection on statistics for biologists contains articles on many of the points above.

Software and code

Policy information about availability of computer code

Data collection	No software used for data collection
Data analysis	stan and python code provided online at https://github.com/jg-you/plant-pollinator-inference as well as under the DOI: 10.5281/zenodo.4759159. Python version: 3.9.4. pystan version: 2.19.1.1

For manuscripts utilizing custom algorithms or software that are central to the research but not yet described in published literature, software must be made available to editors and reviewers. We strongly encourage code deposition in a community repository (e.g. GitHub). See the Nature Research guidelines for submitting code & software for further information.

Data

Policy information about availability of data

All manuscripts must include a data availability statement. This statement should provide the following information, where applicable:
- Accession codes, unique identifiers, or web links for publicly available datasets
- A list of figures that have associated raw data
- A description of any restrictions on data availability

The Mahé visitation data that support the findings of this study are available as supplementary material of Kaiser-Bunbury et al ("Ecosystem restoration strengthens pollination network resilience and function." Nature 542 (2017): 223-227). The data analyzed in Fig. 5 can be downloaded from the Web of Life data base, available at http://www.web-of-life.es, under the network identifier M_PL_019.

Field-specific reporting

Please select the one below that is the best fit for your research. If you are not sure, read the appropriate sections before making your selection.

☐ Life sciences ☐ Behavioural & social sciences ☒ Ecological, evolutionary & environmental sciences

For a reference copy of the document with all sections, see nature.com/documents/nr-reporting-summary-flat.pdf

Ecological, evolutionary & environmental sciences study design

All studies must disclose on these points even when the disclosure is negative.

Study description	This methodological study proposes new tools to analyze existing ecological network data. No original data were gathered so no study needed to be designed.
Research sample	We re-analyzed two existing plant-pollinator data sets, one gathered by Kaiser-Bunbury et al. (Nature, 2017), and one gathered by Inouye and Pyke (Aust. J. Ecol., 1994) re-analyzed by Jordano et al. (Ecol. Lett., 2003).The Kaiser-Bunbury data (Nature, 2017) were gathered in inselbergs (steep-sided monolithic rocky outcroppings) on the tropical granitic island of Mahé, located in the Indian Ocean. It records the visits of pollinator species to all plant species found in each of the eight inselbergs, observed between September 2012 and April 2013 during the island's eight-month-long tropical flowering season. Species visiting flowers were recorded as pollinators if they touched the sexual parts of the flowers within a standard observation window of 30 minutes. Floral abundances were obtained by counting flowers in 1-meter cubes randomly located along transects spanning the inselbergs. The visit data were used to generate 64 data matrices of plant-pollinator interactions, one for each period and location.The Inouye and Pyke (Aust. J. Ecol., 1994) were gathered in the Kosciusko National Park, in New South Wales, Australia, from late December 1983 until the end of March 1984. The paper includes two Appendix Tables, detailing all the observed plant and pollinator species observed, as well as the number visitations of each plant by each pollinator species. It was digitized in Jordano et al. (Ecol. Lett., 2003) and downloaded from www.web-of-life.es
Sampling strategy	We selected the data set of Kaiser-Bunbury et al. (Nature, 2017), as it is among the most well-sampled observational study of plant-pollinator systems. It thus is a best-case scenario of sort for our method. The INO2 data set (Fig. 5) is typical of the data set re-analyzed by Jordano et al. (Ecol. Lett. 2003). It was selected for its particularly small sample size, which is ideal for illustrating how our method performs under harsh sampling conditions. Since our study is not systematic, these two data set are sufficient to support our analysis.
Data collection	Not applicable as we re-used existing data sets.
Timing and spatial scale	Not applicable as we re-used existing data sets.
Data exclusions	No data were excluded from the analysis.
Reproducibility	We verified the convergence of all sampling algorithms by re-running the Monte-Carlo simulations 10 times for each experiment, re-starting the chain if stan provided a convergence error via R-hat > 1.05, or encoutered a divergent point of the posterior distribution. We obtained the same results every time up to small O(sqrt(n)) variations to be expected in Monte Carlo simulations.
Randomization	Not applicable as we re-used existing data sets.
Blinding	Not applicable as we re-used existing data sets.

Did the study involve field work? ☐ Yes ☒ No

Reporting for specific materials, systems and methods

We require information from authors about some types of materials, experimental systems and methods used in many studies. Here, indicate whether each material, system or method listed is relevant to your study. If you are not sure if a list item applies to your research, read the appropriate section before selecting a response.

Materials & experimental systems

n/a	Involved in the study	
☒	☐	Antibodies
☒	☐	Eukaryotic cell lines
☒	☐	Palaeontology and archaeology
☒	☐	Animals and other organisms
☒	☐	Human research participants
☒	☐	Clinical data
☒	☐	Dual use research of concern

Methods

n/a	Involved in the study	
☒	☐	ChIP-seq
☒	☐	Flow cytometry
☒	☐	MRI-based neuroimaging
